# Declarative Representation of Uncertainty in Mathematical Models

**DOI:** 10.1371/journal.pone.0039721

**Published:** 2012-07-03

**Authors:** Andrew K. Miller, Randall D. Britten, Poul M. F. Nielsen

**Affiliations:** 1 Auckland Bioengineering Institute, University of Auckland, Auckland, New Zealand; 2 Department of Engineering Science, Faculty of Engineering, University of Auckland, Auckland, New Zealand; National Research Council of Italy (CNR), Italy

## Abstract

An important aspect of multi-scale modelling is the ability to represent mathematical models in forms that can be exchanged between modellers and tools. While the development of languages like CellML and SBML have provided standardised declarative exchange formats for mathematical models, independent of the algorithm to be applied to the model, to date these standards have not provided a clear mechanism for describing parameter uncertainty. Parameter uncertainty is an inherent feature of many real systems. This uncertainty can result from a number of situations, such as: when measurements include inherent error; when parameters have unknown values and so are replaced by a probability distribution by the modeller; when a model is of an individual from a population, and parameters have unknown values for the individual, but the distribution for the population is known. We present and demonstrate an approach by which uncertainty can be described declaratively in CellML models, by utilising the extension mechanisms provided in CellML. Parameter uncertainty can be described declaratively in terms of either a univariate continuous probability density function or multiple realisations of one variable or several (typically non-independent) variables. We additionally present an extension to SED-ML (the Simulation Experiment Description Markup Language) to describe sampling sensitivity analysis simulation experiments. We demonstrate the usability of the approach by encoding a sample model in the uncertainty markup language, and by developing a software implementation of the uncertainty specification (including the SED-ML extension for sampling sensitivty analyses) in an existing CellML software library, the CellML API implementation. We used the software implementation to run sampling sensitivity analyses over the model to demonstrate that it is possible to run useful simulations on models with uncertainty encoded in this form.

## Introduction

Declarative model representation languages provide a significant opportunity for improving multi-scale modelling workflows, because they cleanly separate the description of the mathematical problem from any algorithmic description, and do so in a way that allows smaller models to be easily composed to build large multi-scale models. Declarative model representation languages are best understood through comparison to imperative languages; imperative languages describe a series of steps taken to perform some computation, while models in declarative languages simply make assertions (as is typically done in descriptions of models in academic literature), leaving the numerical application of those assertions up to software packages. This approach has the important benefit that the same model can be used for multiple purposes. For example, a description of some ordinary differential equations and their initial values (an ODE-IV problem) might be used to render equations for a manuscript, solve the ODE-IV problem numerically to understand the time evolution of the system, be used to compute an analytic Jacobian or analytic solution using another solver package, be used in a sensitivity analysis, and be composed into a large multi-scale model, all without reformulating the model.

A number of declarative mathematical model representation languages exist in the literature; many of them have been developed with particular problem domains in mind. For example, Systems Biology Markup Language, or SBML [1] allows mathematical models to be described, with a focus on systems biology. CellML [2], [3] is an example of a modelling language which has been designed to be domain neutral. The CellML project hosts a repository of CellML models [4] containing, at the time of writing, 557 workspaces, each of which contains one or more related models (mostly drawn from various fields of biology). CellML is also one of the modelling languages selected for use in the European Framework 7 Virtual Physiological Human project. For these reasons, this paper uses CellML as the starting point for representing uncertainty in mathematical models. However, most of what is presented here could be adapted to other declarative languages.

Uncertainty in model parameters can arise from diverse sources. A parameter may have been measured experimentally, yielding information about the value of the parameter, but not an exact value. Often, there may be a statistical model describing prior distributions and the relationship between samples (and the random variables from which they are sampled) and the particular parameterisation used in an experiment; the posterior distribution of the parameters can then be computed either analytically or using numerical methods (such as BUGS, Bayesian Inference Using Gibbs Sampling [5] and subsequent refinements).

Another common source of uncertainty is where there is no experimental data available for a parameter, but due to physical and other constraints, a modeller has an idea of the range of values in which a parameter lies. Modellers will often be able to suggest a subjective probability distribution for the parameter; for example, a modeller who knows that a parameter value must fall in the interval (*a, b*) may postulate, a priori, that the true value is uniformly likely to be any value between *a* and *b*.

A further common source of uncertainty arises when producing models of individuals from a population. Each individual may have a specific fixed value of a parameter, with variation of the parameter across the population; if a particular parameter has not been measured in a particular individual, the parameter is uncertain in an individual-specific model.

ODE-IV problems with uncertain parameters are distinct from stochastic differential equation initial value (SDE-IV) problems. SDE-IV problems contain references to stochastic functions that vary with time, while the class of problem described here describes parameters with a single but unknown true value that holds for all time values.

For uncertainty information to be useful in a declarative model, some representation for the posterior distribution of uncertain parameters is required. We will briefly summarise the existing literature on declaratively representing distributions of uncertain parameters, and then describe an approach for representing these distributions in CellML models.

The BUGS software package includes a declarative language for expressing statistical models [6]. This language can be used to declare stochastic and deterministic relationships between variables (these relationships are referred to as nodes in the paper). Stochastic nodes are described using distribution names taken from a controlled vocabulary, so that only distributions recognised by the software can be specified in the language. Other more recent Bayesian Inference Gibbs Samplers, such as WinBUGS [7], OpenBUGS and JAGS [8] have continued with the controlled vocabulary approach to describing distributions (and in the case of JAGS, providing facilities for more easily adding new distributions to the language). The output from these software packages describes the posterior distribution as a set of samples.

UncertML is a markup language for describing uncertainty using XML. It allows summary statistics about uncertain values to be provided, as well as descriptions of a finite number of distributions from a controlled vocabulary. It also allows distributions in terms of samples, by providing a set of samples (each individual sample called a realisation) drawn from the distribution of a parameter.

We are also aware of a proposal under development as part of the SBML distribution and uncertainty project (http://sbml.org/Community/Wiki/SBML_Level_3_Proposals/Distributions_and_Ranges_Hinxton_Proposal). The approach taken by that project so far provides a wrapper around UncertML to describe uncertainty in terms of realisations and distributions from a controlled vocabulary. This approach would not be adequate for representing uncertainty in CellML models for two major reasons: firstly, it would be incompatible with the principle of using Content MathML to represent mathematical relationships in CellML, and secondly, it would provide an inconsistently low level of expressive power. CellML already has facilities for representing mathematical expressions using Content MathML, and many probability density functions can be represented in closed form without the loss of accuracy arising from using realisations or the loss of expressive power arising from using a controlled vocabulary of distributions.

In this paper, we discuss mechanisms for bringing uncertainty into CellML models. The mechanisms for uncertainty representation presented here fit in naturally with the use of Content MathML to describe models; in addition to allowing distributions to be described using realisations as in UncertML and the SBML distributions and uncertainty project, it allows distributions to be specified by giving the probability density function. We also present an experimental extension to SED-ML (the Simulation Experiment Description Markup Language [9]) for describing sensitivity analysis experiments, and a software implementation of the proposals presented in this paper.

## Methods

### Representing the Information in MathML

CellML makes use of Content MathML [10] to describe mathematical relationships in a structured way. Content MathML provides the *csymbol* operator, which allows external symbols to referenced and included as an operator in a MathML expression.

To support descriptions of uncertain parameters, we introduce three operators to be included in Content MathML expressions. The full definitionURL for these operators is “http://www.cellml.org/uncertainty-1#”, followed by the suffix for the respective operator:

- uncertainParameterWithDistribution takes two arguments. The first argument should be either a variable in the model, or a vector of variables in the model, while the second should be a statistical distribution (built with one of the following two operators). This operator forms an assertion that the variable in the first argument is a random variable with the distribution specified as the second argument.
- distributionFromDensity takes a single argument, which should be a function from a real number to a real number, representing the p.d.f. This function would usually be specified using the MathML lambda constructor.
- distributionFromRealisations takes a single argument, which should be a vector. Each element of the vector represents a realisation of the variable (in which case it should be an expression which evaluates to a scalar value) or variables (in which case each vector element should itself be a vector of expressions which evaluate to a scalar value).

Note that these URIs identify a virtual resource and are recognised by software, but do not refer to any particular document.

CellML requires that all variables and constants are annotated with units (with the possibility that the units are ‘dimensionless’); this rule continues to apply in expressions for realisations and p.d.f.s, with probabilities being dimensionless. This allows software that checks CellML models for units and dimensional consistency to also check descriptions of probability distributions (although such software will still need to be updated to recognise the constructs presented in this paper).

### Adding Uncertainty Support to a DAE-IV Solver

We implemented sampling from a probability density function in the CellML Integration Service, through numerical inversion of the cumulative density function [11] as follows:

Let *X* be the distribution represented by the probability density function *f*; let *x* be the desired sample from *X*. Let *z* be a sample from *Z*, where:
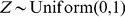
(1)


Note that for a random sampling analysis to be carried out, it is necessary for a source of uniform random or pseudo-random numbers to be available. Most general purpose computing platforms make such uniform random number generators available. For example, POSIX [12] defines the function *random*, which is suitable for use as a uniform random number generator on many platforms. On platforms where no suitable system-provided random number generator is available, algorithms such as the Mersenne Twister [13] can be used to generate a series of values starting from a seed value.

Let *F* be the cumulative density function:
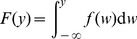
(2)


We perform a change of variable on *w* in the integral to make the limits finite:
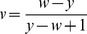
(3)

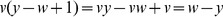
(4)

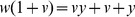
(5)

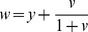
(6)

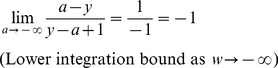
(7)

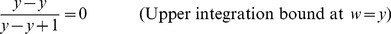
(8)

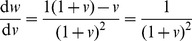
(9)

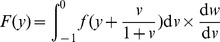
(10)

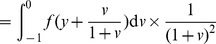
(11)


Using numerical integration, *F*(*y*) can be evaluated from this last form at any *y*. To compute *x* from *z*, we numerically invert *F*(*y*) at *z*, giving:
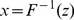
(12)


The numerical integrator to use is selected by the user in the simulation description. The inversion is performed by finding the smallest 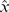 that minimises 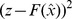 using an existing Levenberg-Marquardt implementation [14]. This approach assumes that *F*(*x*) is a valid cumulative density function, and so is monotonically increasing; the only local minimum of 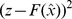 is the global minimum.

This procedure of numerically inverting a function is computationally expensive, but with the models we tested, the cost is still low compared to the numerical integration that follows.

### Details of the Simple Example Model Used

To demonstrate the concepts described above, we coded a simple example model in CellML (Model S1). Our model describes the motion of an object in two spatial dimensions (x and y) experiencing a constant acceleration, and with uncertain initial position and velocity. This model was chosen for its conceptual simplicity.

We created two SED-ML simulation experiment descriptions; one describing a single run of the model, and one describing a sampling sensitivity analysis.

For illustration purposes, we chose the initial position *x* to have a posterior distribution with the components independently normally distributed with a mean of 0 m and a variance of 1 m^2^:
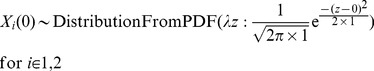
(13)


Note that the 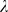 notation 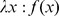, where 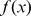 is some expression, is used to define an anonymous function with bound variable *x*.

These distributions were described using the probability density function. Likewise, the x component of the initial velocity was described independently as a sample from the normal distribution with mean 10 ms^−1^ and variance 1 m^2^ s^−2^:
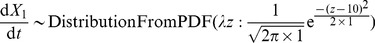
(14)


**Figure 1:**
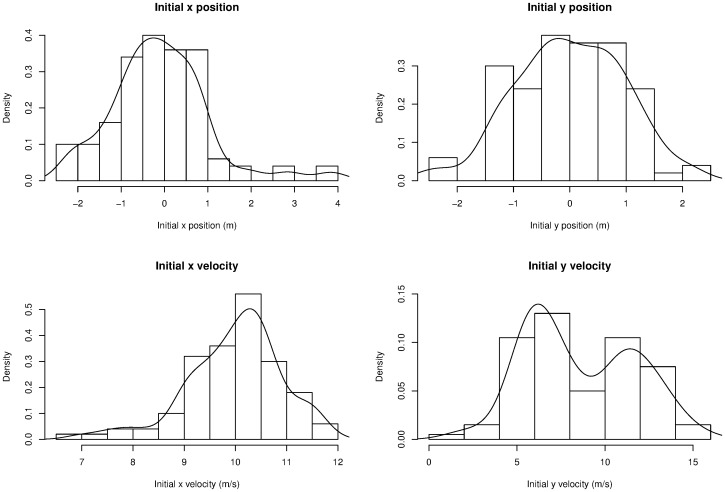
The distribution of the initial position in x and y, and the initial x and y velocity components of the object, shown using both a density histogram and a kernel density plot. Generated using Model S1.

The y component of the initial velocity was described in a different way, to demonstrate the ability to sample values from a set of realisations. To generate the realisations, we created a statistical model. We assumed that the initial y velocity depended on which of two springs was used to propel the object; the spring is selected so that there is a 50% chance of each spring being selected. We further assumed that each spring produced normally distributed initial velocity, with the per-spring mean and per-spring variance unknown. We set the prior distribution for each per-spring mean to be a normal distribution with a mean of 9 and a standard deviation of 0.5, and the prior distribution for the per spring variance to be an exponential distribution with a rate parameter of 20. We additionally provided 40 data values, 20 of which were equal to 6, and 20 of which were equal to 12, with the spring corresponding to each data value unknown. As there is no immediately obvious closed form for the posterior distribution of any additional velocities, it is a good example of where the distributionFromRealisations construct is useful. We used JAGS to produce 1000 samples for the posterior distribution for a velocity (determined independently from the 40 data points), after discarding a burn-in of 1000 samples, and put the retained 1000 samples into the CellML model using distributionFromRealisations. As shown in Figure 1, this produced a bimodal distribution. The representation in CellML is equivalent to:
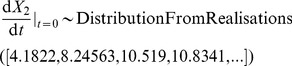
(15)


The remainder of the model describes straightforward equations:
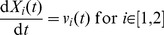
(16)

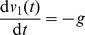
(17)


## Results

### Information to Represent

The CellML specification is intentionally very broad; the underlying philosophy used is to allow a wide range of models to be represented. Some types of software can process all valid CellML models, but other types of software (such as solver software) only support a subset of all models that can be expressed in CellML. It would therefore be helpful if the mechanism for adding uncertainty to CellML allowed the same level of generality to be preserved. For this reason, the approach presented in this paper allows the posterior distribution of uncertain parameters to be represented by specifying the probability density function (p.d.f.), rather than by selection from a controlled vocabulary, thus enabling modellers to express general models. Applications that will only work with a limited number of distributions will then need to recognise distributions from the mathematical form.

It is not always possible to find a closed form for the p.d.f. of the posterior distribution; in these cases, the distribution might only be known from numerical sampling. To support this use case, a mechanism is additionally provided to describe parameter uncertainty using realisations (samples) from the distribution.

### Describing Sensitivity Analysis Simulation Experiments

The scope of modelling languages such as CellML is to represent mathematical models, and so descriptions of how to perform simulation experiments using those models are outside the scope of CellML. SED-ML [9] is an emerging format for describing simulation experiments. The latest publicly available draft of SED-ML only supports one type of simulation, to describe a so called uniform time-course experiment, where a model describing an ODE-IV or differential algebraic equation initial value (DAE-IV) problem is used to find solutions at one or more points between the ‘initial’ bound variable value (at which initial values are provided) and some upper bound.

This type of simulation experiment can be used with models containing uncertain parameters, to find the solution for a single sampled instance of the problem. However, one of the major reasons for describing parameter uncertainty information in the first place is to understand the effects of the parameter uncertainty on the results of the simulation experiment, or in other words, to perform a sensitivity analysis.

There are numerous types of sensitivity analysis possible; one of the simplest and most robust (albeit computationally expensive) is random sampling-based sensitivity analysis [15]. We propose a simple extension to SED-ML to support such simulation experiments, by creating a new type of experiment called a SamplingSensitivityAnalysis. SamplingSensitivityAnalysis extends the existing UniformTimeCourse simulation type, but adds a new attribute, numberOfSamples, to describe the number of random samples to take.

### Implementing Uncertainty in a DAE-IV Solver

As a first step towards validating the proposals presented in this paper, we extended an existing software library for working with CellML models, the CellML API implementation [16], to support simulations of models using these proposals.

We extended the SED-ML Processing Service and SED-ML Running Service within the CellML API to support sampling sensitivity analyses. In addition, we extended the CellML Code Generation Service and CellML Integration Service to allow them to solve DAE-IV problems with uncertainty in the model parameters.

We implemented both univariate and multivariate sampling from a vector of realisations by randomly picking an index from the realisations, so that each index is equally likely to be selected, and assigning the parameter(s) on the left hand side to the value(s) from the selected realisation.

### Results from the Simple Example Model

Figure 1 shows a density plot of the sampled parameters for four different sampled scalars making up the components of the initial position and the initial velocity.

**Figure 2:**
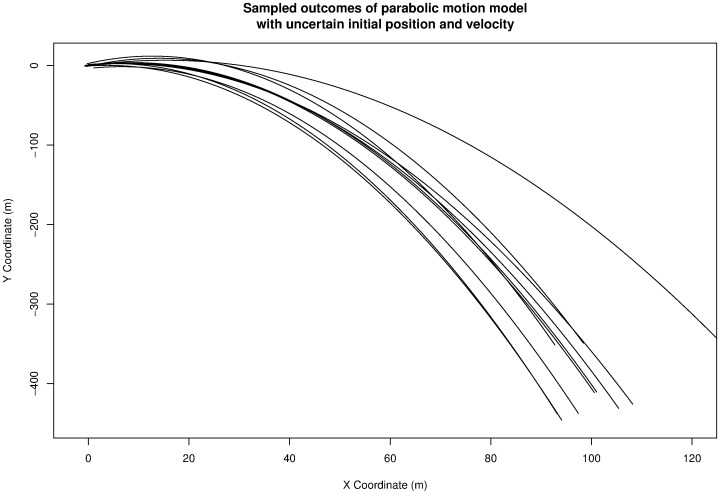
The path of the object in the example model is plotted for ten runs of the model. The path depends on the uncertain parameters. Generated using Model S1.

Figure 2 shows the path taken across ten runs of the model, showing that the initial variation has a significant impact on the path taken and the position after a fixed amount of time (all paths are shown between *t* = 0 s and *t* = 10 s).

**Figure 3:**
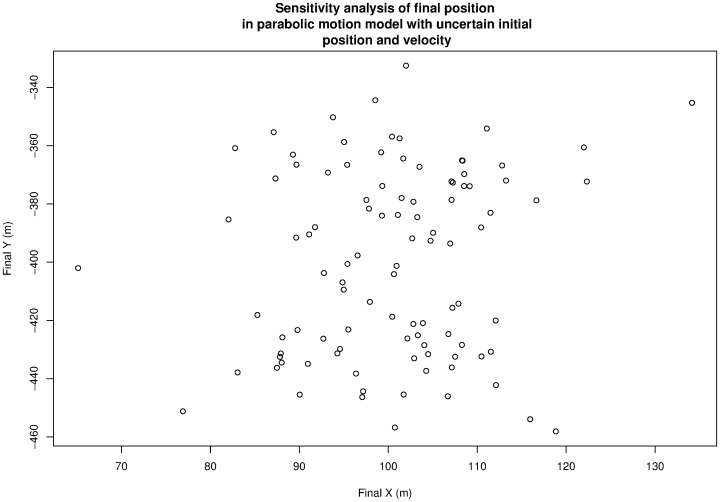
A sensitivity analysis of the example model, showing the position of the object at time 10 s. Generated using Model S1.

Figure 3 shows the output of the sensitivity analysis run, giving the position of the object at 10 s for many different parameters.

The model and simulation descriptions are available online in the CellML model repository [4] at https://models.cellml.org/w/miller/uncertain-starting-parabola. The experimental software implementation of the proposal (including support for both the CellML and SED-ML extensions presented here) has been included in the development branch of the CellML API, available from http://cellml-api.sourceforge.net.

## Discussion

The proposal presented in this paper allows mathematical models coded in CellML to include descriptions of parameter uncertainty. This proposal has been initially demonstrated on a simple physical model, but can be used with CellML models of arbitrary complexity.

The implementation issues around the proposal have been addressed (as discussed in the Methods section), and a software implementation has been produced and tested, demonstrating that the proposal is feasible to implement.

### Future Work

The work presented here is only useful for continuous distributions, where the distribution will almost never be exactly equal to any one particular value, because the p.d.f. is finite everywhere. Such distributions have smooth, differentiable, and monotonically increasing cumulative density functions.

However, mathematical modellers may also need to describe variables sampled from a discrete distribution, and possibly even from distributions which are discrete on some ranges and continuous on others. The mixed case could be handled in MathML using combining constructs such as piecewise to mix discrete and continuous parts, and so the remaining need is to allow random variables with a discrete distribution to be represented. The representation presented here could be extended to allow discrete distributions by adding a new MathML *csymbol* for describing a probability distribution using a probability mass function (p.m.f.). Such discrete random variables would require a different numerical sampling algorithm. Determining the discrete values at which the p.m.f. is defined purely numerically is a difficult problem, but with a CellML model, it is possible to combine automated symbolic analysis with numerical analysis. This approach would be feasible for the class of discrete problems where a piecewise is used to ensure the probability is zero except for cases which consist of some transformation of finite sets. In such cases, the finite set could be computed by applying the transformation in the piecewise case condition. A numerical algorithm would take a sample from the uniform (0,1) distribution, compute the probability at each member of the finite set, and compute a cumulative sum of the probabilities until the sampled value was exceeded. While this would not cover every possible p.m.f., it would most likely be general enough to support most cases needed in practice.

The approach taken in this work does not allow multivariate probability density functions to be described using probability density functions. This limitation can sometimes be bypassed by describing a joint distribution as a univariate marginal distribution and a series of conditional distributions for the remaining random variables. Current ratified versions of CellML do not provide a mechanism to specify that a variable has a type other than real, and so software that processes CellML does not typically need to support vector mathematics. However, if future versions of CellML did allow variable types such as ‘vector of real numbers’ to be specified, it would make the inclusion of multivariate probability density functions more natural.

The use of probability distribution functions to describe distributions fits cleanly with the design of CellML, but it represents a significantly different approach to the controlled vocabulary approach taken in UncertML. An important area of future work is to investigate the interconversion between uncertainty specifications in UncertML, and uncertainty specifications using the approach discussed here. Conversion from UncertML to the approach discussed here should be relatively simple for most distributions, because it is simply a matter of substituting the form for the corresponding probability density function. Due to the higher level of generality of the approach presented here, conversion in the reverse direction will not always be possible. However, it would be possible to identify p.d.f.s for particular well-known forms of the distributions supported by UncertML, and convert those forms into the corresponding Content MathML.

The methodology presented in this paper represents probability distribution functions in a declarative form, which admits the possibility of both analytic and numerical analysis, as well as approaches that combine automatic analytic manipulation with numerical solution. The work presented in this paper primarily relies on numerical analysis. In some cases, however, it may be significantly more efficient to perform analytic work on the p.d.f. (and possibly the entire DAE-IV system) prior to any numerical analysis. In the case where the inverse of the c.d.f. has a closed form, automated symbolic manipulation could allow this closed form to be computed analytically.

A great deal of theoretical work has been published on how to efficiently sample from particular probability distributions; for example, the normal distribution [17] and the gamma distribution [18]. The work presented in this paper does not currently use these optimised algorithms because of the focus on generality. However, future work could preserve support for the general case, while detecting p.d.f.s corresponding to a distribution for which a more efficient algorithm is available.

In addition, there are a number of alternative general numerical algorithms for sampling from a continuous probability density function. The rejection method [19] allows for sampling directly from the probability density function, using two uniform random samples. The rejection method requires upper and lower bounds on both the density function and the random variable being sampled, and so automated symbolic analysis to determine these bounds would be required for an efficient rejection based method.

As is common with numerical integration problems, the presence of step-wise discontinuities can cause problems for numerical solvers. Consider the case of a uniform distribution; the probability density function is zero outside a certain range of random variable values, and a constant value inside the range. The only way that a numerical integration algorithm can determine that the function is not zero everywhere is if it happens to find a point inside the range. Numerical integration algorithms can only take a finite number of samples, so if the range is very small, it may be missed entirely by the numerical integrator. This problem can already occur in DAE-IV problems (where it is common to want to numerically integrate the DAE-IV system over a variable such as time). Generally, modellers can work around the problem by adjusting the numerical parameters to ensure that the maximum step size used by the numerical algorithm is small enough to ensure that the algorithm will find the step. However, a more general solution could be to analytically detect piecewise expressions, and numerically identify the boundary of a transition between piecewise cases, and ensure that the solver carries out a step in every piecewise case. Note, however, that a similar issue can arise with narrow normal distributions, because outside a certain range, the probability density is so small that it is represented by the floating point number zero.

## Supporting Information

Model S1 **The simple example model used to demonstrate how uncertainty can be represented using CellML.**
(ZIP)
